# Traumatic injuries in pregnant women: anesthesiology aspects

**DOI:** 10.5249/jivr.v3i2.103

**Published:** 2011-07

**Authors:** Naser Yeganeh

**Affiliations:** ^a^Department of Anesthesiology, Imam Reza Hospital, Kermanshah, University of Medical Sciences, Kermanshah, Iran.

Among the most common predisposing factors that increase the anesthetic-related risks in the management of  traumatic injuries are; patients presenting with depressed level of consciousness, full stomach, hypothermia, major blood loss, and screening positive for alcohol or drug abuse. Such patients, therefore, are more likely to respond differently to anesthetic agents. In reference to the current case,^1^we do not have any data to show patient’s blood pressure, heart rate, respiratory rate and urine output. However, according to her medical chart, we do know that her estimated blood lost was 10-15%. In such circumstances it seems logical to start the patient with intravascular fluid replacement with crystalloids infusion.^2^In all cases of multiple traumatic injuries, patients are at high risk for cervical spine trauma even when the related signs or symptoms are not present. This is especially important to consider when patient is transported by ambulance, or is transferred between different diagnostic wards, or during the time when rapid sequence induction is mandatory in anesthesia induction time.^3,4^Safety measures and precautions that are recommended for such cases include; use of appropriately sized Philadelphia collar, placing sand bags on each side of the patient’s head and neck, and resting the patient on a hard board with the forehead taped and secured to the board. These considerations however are often neglected in our practice. Other major risk factors in the management of multiple traumas include; tension pneumothorax, occult blood loss and occult long bone fractures, all requiring vigilance of emergency department physicians and personnel.

For anesthesiologists, the choice of selecting a particular anesthetic agent is not as crucial as is deciding what does is more appropriate to use. Thiopental and propofol are appropriate drugs for trauma patients with minor blood loss (below 15%) if given in reduced doses.^5^Ketamine may be used in the hypovolemic patients. Of note, however, is that occasionally trauma patients, while under emergency care, may endure intracranial injuries with no prominent symptoms. Synthetic opioids, such as fentanil and sufentanil are preferable options for maintenance of anesthesia, but when used alone they carry the risk of awareness during anesthesia, which is quite prevalent in trauma patients. Therefore, anesthesiologist are recommended to use these drugs with some adjutants like benzodiazepines, scopolamine and/or nitrous oxide, if possible.^6^

Furthermore, management of pregnant women with traumatic injuries calls for immediate consultation and coordinated care with an obstetrician. This is to ascertain the viability and proper care of the fetus. My question in reference to the present case is why the pregnancy was terminated by hysterectomy despite the fact that the fetus was alive and active and had normal fetal heart rates. Also, there was enough amniotic fluid; and there was no laceration or hematoma to the placenta or other solid organs. It is advisable, however, that some anesthetic agents should be avoided in the first trimester of pregnancy to guard against teratogenicity, such as chlordiazepoxide and nitrous oxide.^7^In hemodynamically unstable trauma patients invasive hemodynamic monitoring is mandatory. This usually consists of continuous intra-arterial blood pressure measurement and central vein cannulation. In addition, patient’s temperature should be monitored regularly and in cases where patient's temperature is below 36ºC active warming should be applied.

The following care and precautions should be considered while the patient is in the postoperative care unit:, providing sufficient analgesia, balancing of intravascular volume and acid-base status, blood products replacement, identifying strategy for pulmonary emboli prevention due to the long bones fractures and prolonged bed rest, identifying methods of preventing hypothermia, and performing thorough physical exams for detection of any hidden injuries. Presence of lung contusion due to blunt chest trauma is a warning sign that the patient may need postoperative mechanical ventilation and intensive care therapy for several days. Continuous monitoring of the fetus heart rate for detecting fetal distress and unexpected abortion is also highly advised during the management of such patient.
